# Leaving-Group Effects in S_N_2@P: Potential Energy Surface and Dynamics of the F**^–^** + PH_2_I Reaction Compared to Its Cl**^–^**‑Leaving-Group Analogue

**DOI:** 10.1021/acsphyschemau.6c00016

**Published:** 2026-04-07

**Authors:** Laura C. Erdei, Dóra Papp, Gábor Czakó

**Affiliations:** † MTA-SZTE Lendület “Momentum” Computational Reaction Dynamics Research Group, Interdisciplinary Excellence Centre and Department of Physical Chemistry and Materials Science, Institute of Chemistry, 37442 University of Szeged, Rerrich Béla tér 1, Szeged H-6720, Hungary; ‡ Department of Physical Chemistry and Materials Science, Institute of Chemistry, University of Szeged, Rerrich Béla tér 1, Szeged H-6720, Hungary

**Keywords:** S_N_2@P, front-side attack, retention, halogen-bonded complex, indirect dynamics, potential energy surface, quasi-classical trajectory simulation

## Abstract

We investigate the dynamics of the F^–^ + PH_2_I reaction using the quasi-classical trajectory method on a newly developed ab initio analytical potential energy surface, with a special focus on stereoselectivity and leaving-group effects by comparing to previous F^–^ + PH_2_Cl results. Besides the overall more exothermic energy profile, the S_N_2 pathway of the F^–^ + PH_2_I reaction is found to be more indirect, with vibrationally highly excited products, and the retention of configuration more relevant due to a much more stable halogen-bonded prereaction complex supporting front-side attack, with respect to the Cl^–^-leaving-group process. Several further retention mechanisms of the title reaction are identified, as well, interestingly excluding multi-inversion, recently discovered at the nitrogen-center, while inversion mainly occurs through stripping. The proton-transfer channel, favoring also the stripping mechanism, is a primarily direct process with less internal excitation in the products, similarly distributed between rotation and vibration.

## Graphical abstract

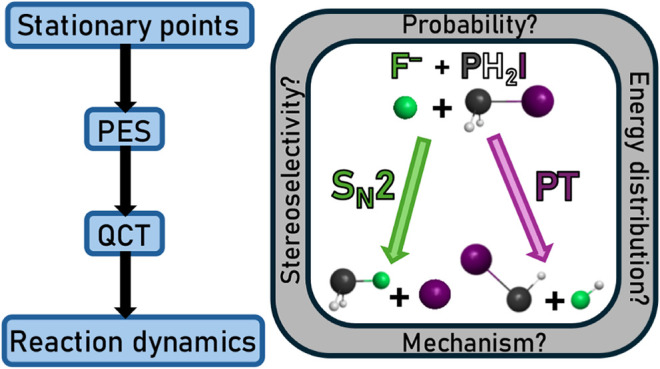

## Introduction

Bimolecular nucleophilic substitution (S_N_2) is one of the most essential reaction families in chemistry, with S_N_2 at carbon center (S_N_2@C) being extensively studied in the past decades.^1–37^ From the 2000s, advances in both experimental^10,15,17,24,25,33^ and theoretical^11,13–15,17,19,20,23–25,29,33,36^ techniques led to a particularly successful interplay between the two fields in studying S_N_2@C reactions. On the theoretical part, several reaction mechanisms beyond the long-known Walden inversion^38^ and front-side attack^39^ have been revealed by dynamics simulations, mainly based on the research of Hase and coworkers:^10–12,15,18^ the complex-mediated roundabout^10^ or H-bond-formation^11^ pathways, as well as the direct stripping and rebound reaction routes.^11^ They applied the so-called direct dynamics approach, where the gradients of the potential energy surface (PES) are computed on-the-fly, along with the quasi-classical trajectory (QCT) method. In the past decade, our group started to utilize a different approach to model S_N_2 reactions:^14,21^ a full-dimensional analytical function, representing the PES, is fitted to a few tens of thousands of energy points obtained from high-level ab initio calculations, and the gradients of the potential energy are determined by differentiating the above analytical function. This way, instead of a few hundreds of QCTs requiring millions of on-the-fly gradient computations, we are able to run several hundreds of thousands of trajectories based on these analytical PESs, which are usually fitted on coupled-cluster/triple-ζ-quality energy points. Our approach made possible to uncover novel, even low-probability, reaction routes in S_N_2@C, such as double inversion^16^ and oxide-ion substitution,^26^ and to distinguish between S_N_2 and bimolecular elimination (E2).^25^

Not only S_N_2@C, but silicon-, nitrogen-, and phosphorus-centered S_N_2 reactions have also been studied; however, much scarcer literature is available regarding the central-atom effects in S_N_2.^40–62^ The pioneering work, based on density functional theory (DFT) stationary-point calculations, of Bickelhaupt and coworkers showed interesting features of third-row-central-atom S_N_2 reactions: Walden inversion usually does not occur through a transition state (TS) on the PES but via a stable minimum, resulting in a single-well profile of these reactive PESs, in sharp contrast to S_N_2@C.^40,41,44,58,59^ In addition, front-side attack, leading to configuration retention around the central atom, has been shown to have a barrier submerged below the reactant asymptote at Si- and P-center.^40,41,44,58,59^ In 2021, the analytical-PES-based QCT approach revealed a novel mechanism in the F^–^+ NH_2_Cl reaction called multi-inversion, which involves repeated inversion through a [FH···NHCl]^−^ complex at lower collision energies, resulting in undermined stereoselectivity, a major difference from S_N_2@C.^55^ Later, we have also developed a PES for the F^–^ + PH_2_Cl reaction to further study the effect of the central atom in S_N_2 reactions, especially on their stereodynamics, and found that retention of the initial configuration is more relevant at higher collision energies, in contrast to F^–^ + NH_2_Cl.^61^ A systematic high-level ab initio stationary-point search including many product channels of the X^–^ + PH_2_Y [X, Y = F, Cl, Br, I] reactions suggested a broad variety of possible mechanisms for S_N_2@P.^62^ Besides, dynamics studies on S_N_2@Si have recently revealed compromised stereospecificity, as well.^47,48^

Investigating the effect of the leaving group at phosphorus center is a direct step forward, especially because it is motivated by a joint experimental-theoretical S_N_2@C study that unveiled a drastic shift to indirect dynamics at C center when the Cl^–^ leaving group was replaced by I^–^ in the F^–^ + CH_3_Cl reaction.^17^ Later, it has been shown that this effect is caused by a deep halogen-bonded entrance channel minimum in the case of the I^–^ leaving ion.^20^ Varying the nucleophile, the leaving group and other substituents at tri- and tetracoordinated phosphorus centers in the DFT stationary-point computations of Bickelhaupt and coworkers has proven to strongly affect the stability of the transition species or even the shape of the energy profile of both back- and front-side attack.^44,58,59^

In the present article, we report a full-dimensional analytical ab initio PES for the F^–^ + PH_2_I reaction, developed using the Robosurfer program package,^63^ and explore its dynamics by means of QCT simulations. We follow the configurational changes around the central atom, along with the energy flow during the reaction. We also identify different mechanisms based on attacking- and scattering-angle distributions of the reactants and the products, respectively, and by visual investigation as well. Then, we compare our dynamics results with those previously obtained for the F^–^ + PH_2_Cl reaction^61^ to study the impact of the leaving group in the S_N_2@P reactions and also to the F^–^ + NH_2_Cl process^55^ to extend our investigations on central-atom effects in S_N_2.

## Computational Details

As a first step of investigating the dynamics of the title reaction, we generate randomly displaced geometries based on the CCSD­(T)-F12b/aug-cc-pVTZ optimized structures of the stationary points of the F^–^ + PH_2_I reaction, which have been previously determined.^62^ The displacements of the Cartesian coordinates fall in the 0.0–0.4 Å interval. In the case of the reactants and the products, the smaller fragment is scattered around the center of mass (COM) of the polyatomic fragment in the 2.5–15.0 Å distance range. We create 500–500 geometries for the transition states and the midreaction minima and 1000–1000 geometries for the reactants and products, respectively. Then, we compute the energies of the randomly generated points, for which we use a Brueckner-type composite method, proven to be useful in eliminating the breakdown of standard CCSD­(T) at geometries far from equilibrium:^23^

1 CCSD‐F12b/aug‐cc‐pVTZ+BCCD(T)/aug‐cc‐pVTZ−BCCD/aug‐cc‐pVTZ

In order to overcome Hartree–Fock convergence issues, we utilize the ManyHF scheme^64^ which obtains the lowest-energy Hartree–Fock solution based on energy computations starting from various guess orbitals. All quantum chemical computations are carried out using the Molpro software package.^65^ From the initial data set consisting of 14785 energies and geometries, we start the construction of the PES using the Robosurfer program package^63^ and the above composite method. We note that the improvement of the PES takes significantly more time than we usually experience, as the number of unphysical trajectories decreases very slowly during development. The reason behind this might be the electronic-structure challenges that phosphorus as a central atom imposes, e.g., the weak P–H bonds or the larger core of the central atom. For fitting the analytical function representing the PES, we use permutationally invariant polynomials^66^ of the Morse-like variables *y*_*ij*_ = exp­(−*r*_*ij*_/*a*) of interatomic distances *r*_*ij*_, where the *a* constant, set to 3.0 bohr in the case of this ionic reaction, regulates the decay of the exponential function. The highest polynomial order used for this PES is 6, resulting in 4285 coefficients. A weight, described in ref. 67, with parameters *E*_SlopeStart_ = 10 kcal/mol, *E*_SlopeEnd_ = 30 kcal/mol, and *w*_SlopeEnd_ = 0.5, for each potential energy of the fitting set is applied to enhance the relevance of the chemically interesting energy region. For target accuracy of the fit, we set 0.5 kcal/mol for the first 460 Robosurfer iterations and then 1.0, 1.5, and 2.0 kcal/mol for the next 376, 745, and 796 iterations, respectively. The development is carried out with the following number of iterations at the following collision energies: 232, 133, 559, 1124, 182, and 147 iterations with 1.0, 10.0, 20.0, 30.0, 40.0, and 50.0 kcal/mol collision energy, respectively. The development takes altogether 2377 iterations, and the final PES contains 52662 points. The root-mean-square (RMS) error in the energy region 80 kcal/mol above the global minimum (Walden min) is 1.71 kcal/mol, which is somewhat larger than usual, and can probably be attributed to the more extended configurational space that should be covered for the reaction due to the weak P–H bonds.

With the final PES at hand, we run QCT simulations at seven collision energies: 0.9, 6.9, 10.0, 20.0, 30.0, 40.0, and 46.1 kcal/mol, to match those used in our F^–^ + NH_2_Cl^55^ and PH_2_Cl^61^ reaction studies. The initial orientation of the reactants is random in each trajectory, and standard normal mode sampling^68^ is used to set the zero-point energy (ZPE) of the PH_2_I molecule. The impact parameter *b*, i.e., the perpendicular distance of the velocity vectors of the reactants, varies between 0.0–30.0 bohr at the lowest collision energy and 0.0–17.0 bohr at higher energies with 1.0 bohr step size. We run 5000 trajectories at each *b* value, which means altogether approximately 700,000 trajectories. The trajectories are propagated with a 0.0726 fs time step, and the simulations stop when the largest atom–atom distance gets 1 bohr larger than the largest initial one.

For the two main reaction channels (S_N_2 and proton transfer (PT)), we calculate the integral cross sections (ICSs) as a *b*-weighted numerical integration of the *P*(*b*) reaction probabilities at each collision energy. For the ICS values of the PT channel, three different ZPE restrictions are applied: (1) SOFT: the sum of the classical vibrational energies of the products (HF + PHI^–^) must be larger than the sum of the ZPE of PHI^–^ and the ZPE of HF, (2) HARD: the above restrictions are set separately for each of the products, and (3) PHI^–^ ZPE: the constraint is set only for PHI^–^. The scattering angle distributions of the products are defined as binning the cosine of the included angle (θ) of the relative velocity vectors of the products and the reactants into 10 equidistant bins from −1 to +1. Cos­(θ) = −1 (θ = 180°) indicates backward scattering of the PH_2_F and HF products. The attacking angle (α) is defined as the initial included angle of the velocity vector of the COM of the PH_2_I reactant and the vector pointing from phosphorus to iodine, and its cosine is also binned into 10 equidistant bins. For the S_N_2 channel and the nonreactive trajectories, we distinguish between retained and inverted configurations. Retention happens when the scalar product of the normal vector of the HPH plane and the PX (X = I, F) vector has the same sign as in the initial reactant configuration; otherwise, inversion occurs.

## Results and Discussion

We develop a full-dimensional analytical ab initio PES for the F^–^ + PH_2_I reaction and study its dynamics, with a special focus on stereoselectivity, by running quasi-classical trajectories using the novel PES. The development of the PES has turned out to be rather problematic, most likely due to the weak central-atom-ligand bond that requires advanced electronic-structure solutions and an increased flexibility of the fitting function. However, by applying the ManyHF-^64^ and the Brueckner-corrected composite^23^ electronic-structure methods, along with the Robosurfer automatic PES-developer program package,^63^ we have been able to reach appropriate accuracy for dynamics simulations.

The two main channels of the above ion–molecule reaction are the S_N_2 route leading to I^–^ + PH_2_F and proton transfer with products HF and PHI^–^. The S_N_2 reaction may proceed via Walden inversion, as well as front-side (FS) attack, resulting in retention of the initial configuration of PH_2_I, or even via the recently discovered multi-inversion mechanism,^55^ prevalent in the F^–^ + NH_2_Cl reaction,^55^ involving repeated inversional motion in the entrance channel through an H-bonded complex and the corresponding TS. The relevant stationary-point geometries along with the classical and adiabatic (zero-point-energy-corrected) relative energies, obtained both with high-level ab initio methods (benchmark)^62^ and on the analytical PES, of the title reaction are shown in Figure 1. As seen, all of the different pathways lack both thermodynamic and kinetic barriers, as both the S_N_2 and PT channels are exothermic, and all transition states have energies submerged below the reactants. The barrier heights corresponding to the self-inversion of the reactant and product molecules are substantial, just as for the F^–^ + PH_2_Cl reaction,^61,62^ while the hydrogen-bond formed between the attacking F^–^ ion and the PH_2_I molecule (or rather between HF and PHI^–^) significantly lowers the self-inversion barrier of the reactant, similarly to the case of the Cl^–^ leaving group. Since studying the dynamics of the F^–^ + PH_2_Cl reaction,^61^ we have identified two novel stationary points for front-side attack: a planar-structured FS TS and an FS postmin.^62^ A prereaction DI TS (DI stands for double inversion due to traditional reasons^16^) has also been identified during the present study, for which we have calculated benchmark ab initio energies using the same level of theory as in ref. 62 In fact, the relative energies of the stationary points of the title reaction are around 5–6 kcal/mol lower than the ones corresponding to the geometries with the Cl^–^ leaving group, except for the FS premin and FS postmin structures, which lie 20 and 10 kcal/mol lower with respect to those in the F^–^ + PH_2_Cl reaction, respectively. The nonreactively oriented FS premin geometry has been shown to drastically impact the dynamics of an S_N_2 reaction at carbon center.^17,20^ Here, the PT reaction is expected to be somewhat less competitive with S_N_2 due to the larger energy gap between the two product asymptotes compared to the F^–^ + PH_2_Cl reaction. The deep Walden minimum with extremely product-like geometry, similar to the one with a Cl^–^ leaving group, is also the global minimum of the PES of the title reaction.

**Figure 1:**
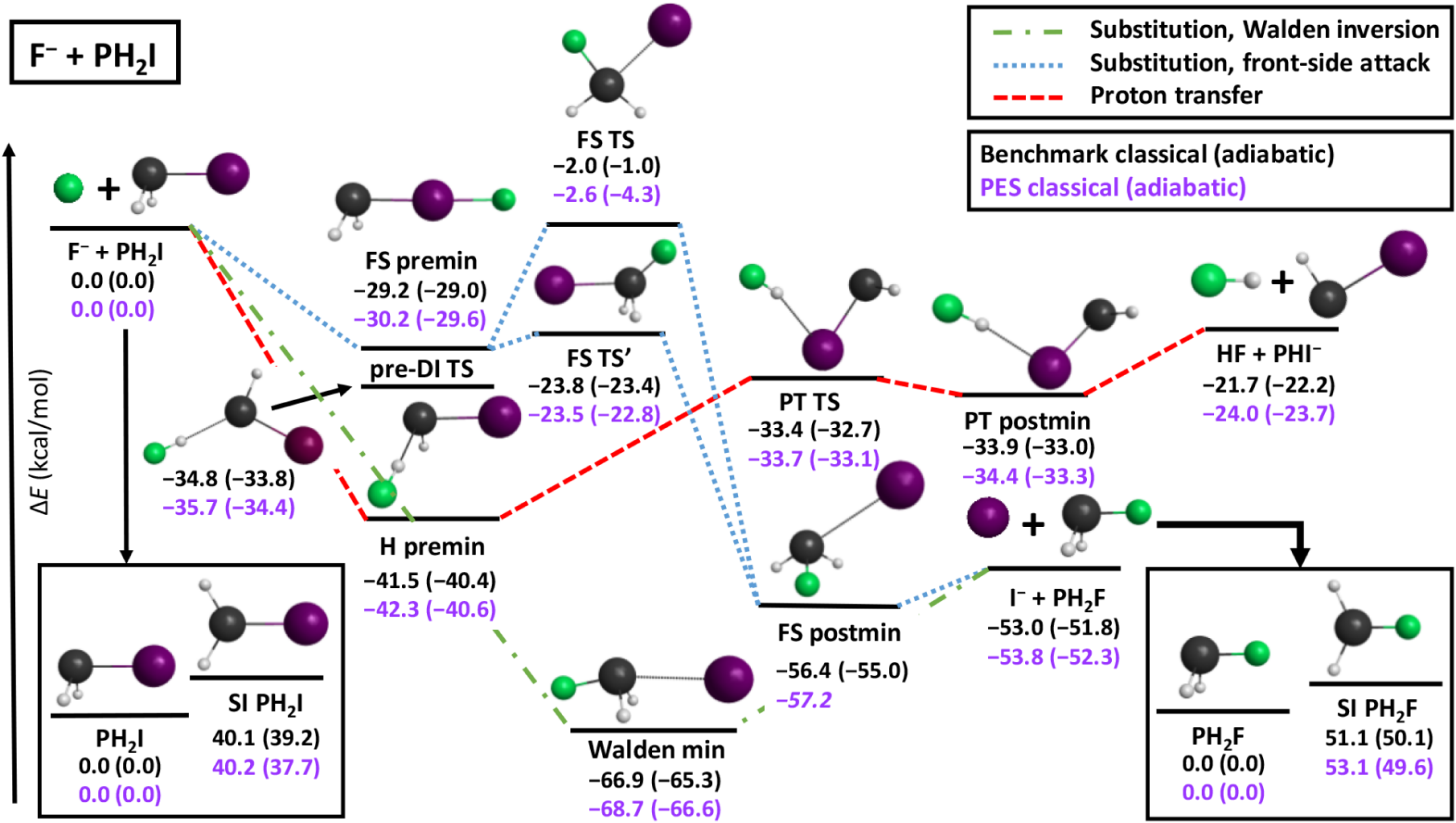
Geometries along with benchmark classical and adiabatic relative energies^62^ of the relevant stationary points of the F^–^ + PH_2_I reaction compared to the energies obtained on the present PES (italics denote single-point energy).

Integral cross sections (ICSs) of the S_N_2 and PT channels of the F^–^ + PH_2_I reaction as a function of the collision energy are presented in Figure 2. In panel A, we show S_N_2 and PT ICSs, the latter ones determined also by the HARD ZPE constraint, meaning that the classical vibrational energy of each product molecule must be larger than its ZPE. Except for the lowest collision energy, the PT channel dominates the reaction, even using the HARD constraint, which is not expected based on the reactant energies and submerged barriers. However, the deep FS premin, owing to its nonreactive arrangement, induces a rather significant inhibition to the S_N_2 reaction, which might be the reason behind the PT dominance. In addition, the system, as also seen in the case of the Cl^–^ leaving group, may tend to avoid the deep Walden minimum at higher collision energies, which can contribute to the decreasing S_N_2 reactivity as well. From panel B, it is clear that the SOFT and PHI^–^-ZPE restrictions have practically no effect on the reactivity of proton transfer, and the HARD constraint reduces the ICS only slightly. This can be explained by the substantial exothermicity of the PT route, which can compensate for the energy of the potentially ZPE-violating products. This is in contrast to what has been found for the F^–^ + PH_2_Cl reaction, where both the SOFT and HARD restrictions had significant effects on reactivity, in accord with the higher (less exothermic) PT reaction energy of the Cl^–^-leaving-group reaction. Panel C of Figure 2 shows the S_N_2 cross section decomposed into inversion and retention reactivities and indicates that products with inverted configuration dominate the reaction at low energies; however, retention becomes more significant as collision energy rises. This is also reflected in panel D, revealing an increasing ratio of retention as collision energy increases. The retention/inversion ratio curve has a similar shape and magnitude as observed in the case of the Cl^–^ leaving group,^61^ although the fraction of retention starts to increase at a lower energy. This difference can be attributed to the deeper halogen-bonded FS premin well of the title reaction, which has a nonreactive orientation for Walden inversion, but at the same time, it can be more favorable for the front-side attack path leading to products with retained configuration. Therefore, as collision energy increases, and thus the system starts to avoid the deep Walden minimum,^61^ FS premin may also drive the reaction more and more to the front-side pathway. In the case of nonreactive trajectories, we find that the ratio of the inverted PH_2_I “products” is negligible, as shown in panels E and F of Figure 2, suggesting that collision-induced inversion is not important at all. This is in sharp contrast to what we have observed at N-center, where induced inversion played a crucial role in the dynamics,^55^ and also to what we might expect from the energetics of Figure 1 because, despite the high self-inversion (SI) barriers, the inversion barrier of the H-bonded complex is only 7 kcal/mol. Induced, and consequently multi-inversion, is also less significant in the F^–^ + PH_2_Cl reaction relative to F^–^ + NH_2_Cl;^55,61^ thus, phosphorus center seems not to favor this mechanism, probably due to the deep Walden-inversion well competing successfully with the H-bonded minimum and inversion TS. Moreover, the large I ligand in the title reaction appears to further hinder induced inversion.

**Figure 2:**
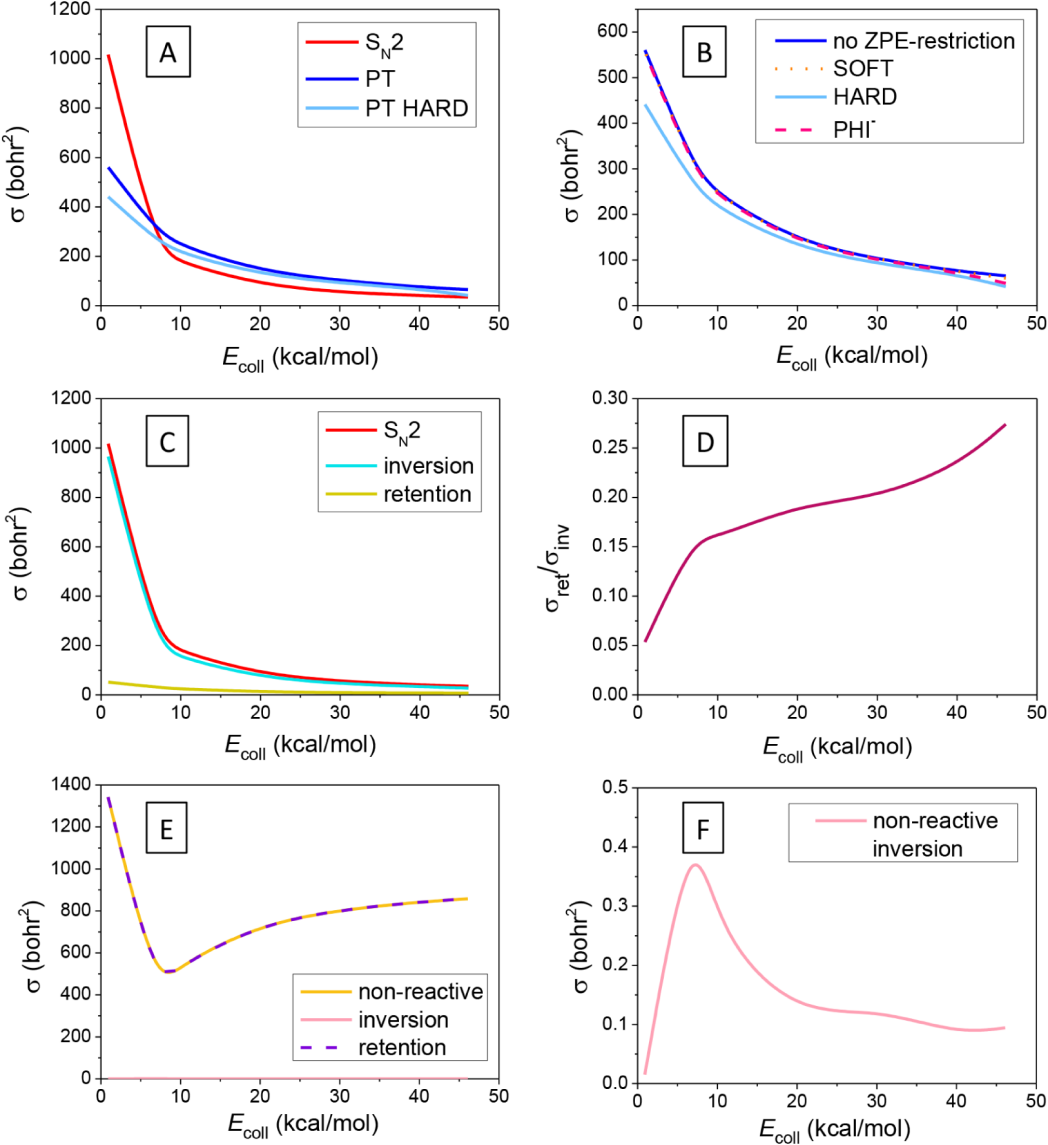
**A**: Integral cross sections of the S_N_2 and PT channels of the F^–^ + PH_2_I reaction as a function of the collision energy. **B**: Integral cross sections of the PT channel of the F^–^ + PH_2_I reaction as a function of the collision energy obtained with different ZPE restrictions (see Computational details). **C**: Integral cross sections of the S_N_2 channel of the F^–^ + PH_2_I reaction as a function of the collision energy decomposed based on the configuration of the PH_2_F product. **D**: Ratio of the inversion and retention integral cross sections of the S_N_2 channel of the F^–^ + PH_2_I reaction as a function of the collision energy. **E**: Integral cross sections obtained from the nonreactive trajectories of the F^–^ + PH_2_I system as a function of the collision energy, decomposed based on the configuration of the PH_2_I “product”. **F**: Integral cross sections of inversion obtained from the nonreactive trajectories of the F^–^ + PH_2_I system as a function of the collision energy.

Opacity functions (reaction probabilities as a function of the impact parameter (*b*)), along with scattering and attacking angle distributions of the S_N_2 channel of the title reaction, also divided based on product configuration, are depicted in Figure 3. From all panels of Figure 3, we can see that the curves corresponding to the lowest collision energy separate distinctly from the others (except the scattering angle distribution corresponding to inversion), indicating a special behavior of the reaction at near-zero collision energy. Along with the decreasing inversion probabilities with increasing collision energy, the possibility of retained-configuration product formation increases at higher collision energies. It is also clear that at lower energies, inversion determines the dynamical behavior of the S_N_2 channel. These observations are in accordance with the retention-inversion ICS ratio of Figure 2(D). The maximum value of the impact parameter, where the S_N_2 probability vanishes, is the largest (27 bohr) at the lowest collision energy and decreases significantly from 18 bohr to 11–12 bohr with increasing collision energy. While inversion probabilities show a preference for large-impact-parameter collisions, the retention reaction path prefers small *b* values. This results in forward-peaking inversion scattering-angle distributions, a clear sign of the stripping mechanism, prevalent at large *b*, where the attacking F^–^ ion strips away the PH_2_ group while keeping its initial direction. Small backward-scattering peaks can also be observed in the case of inversion, suggesting a minor relevance of the direct rebound mechanism occurring at small impact parameters. Interestingly, inversion scattering-angle distributions exhibit no collision-energy dependence. In the case of retention trajectories, we see more isotropic distributions with minor sideways peaks and forward preference at higher energies. Isotropicity may be a sign of complex formation, e.g., FS premin, where the information on the incident directions is not preserved during the lifetime of the complex, whereas sideways scattering indicates the increasing preference for front-side attack at high collision energies, reflecting the characteristics of the FS TS geometries. Surprisingly, the retention distribution is less isotropic at the lowest collision energy but also less forward-scattered than inversion, in accord with the more monotonous decrease of the retention opacity function.

**Figure 3:**
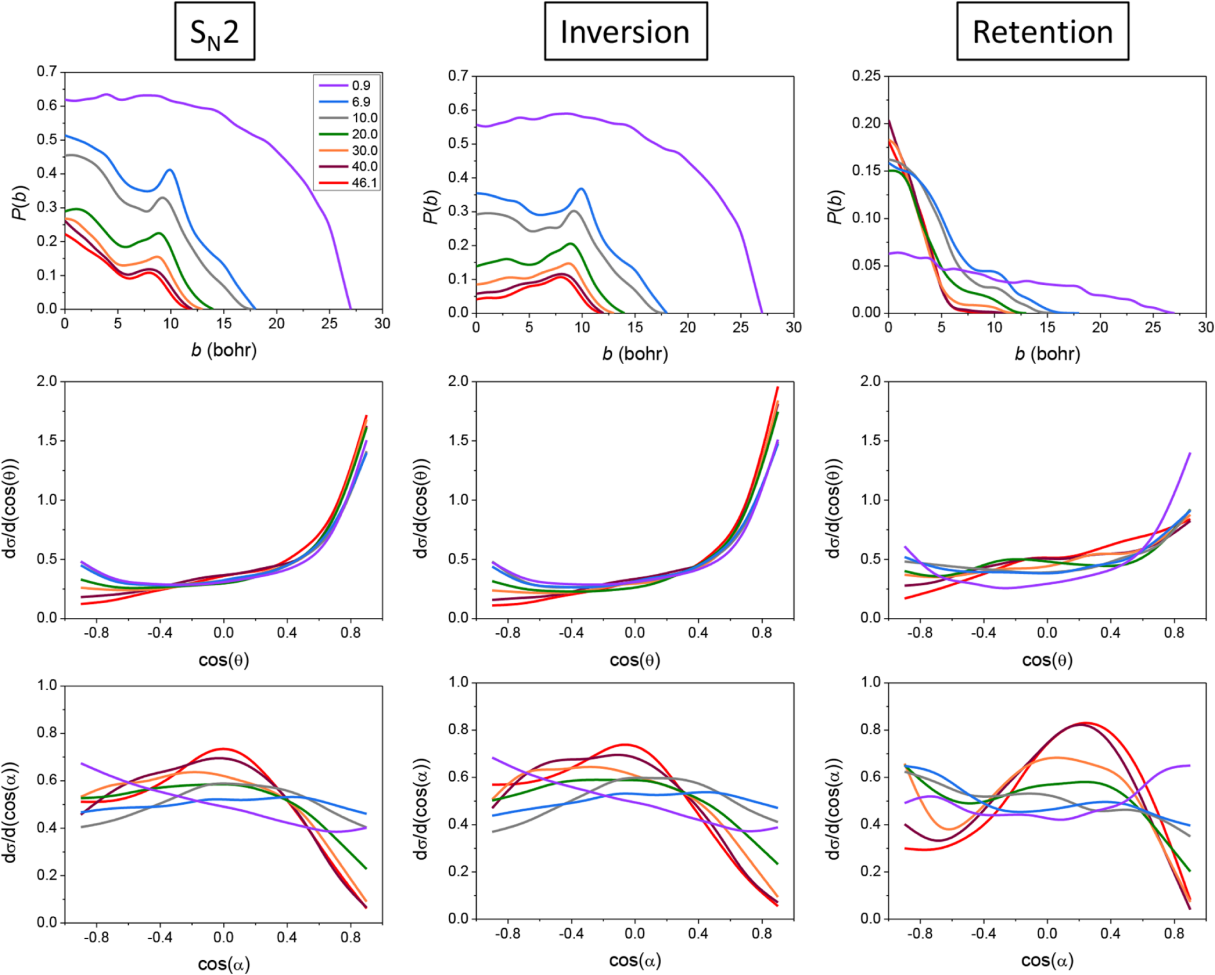
Opacity functions (upper row), scattering angle distributions (middle row), and attacking angle distributions (lower row) of the S_N_2 channel of the F^–^ + PH_2_I reaction divided into inversion (2nd column) and retention (3rd column) at different collision energies (kcal/mol) denoted with different colors.

As to the initial attacking angles, we observe a preference for larger angles (90° and above), especially at the lowest collision energy, which signals the usual backside-attack mechanism of Walden inversion. The maxima around 90° become sharper as the collision energy increases, indicating the prevalence of the stripping mechanism, which can also be favored by sideways attack at large impact parameters, which is more relevant at higher energies. We also see isotropic characteristics at lower energies because the small momentum of the reactants helps to promote attractive interactions, and thus, many reactive collisions can occur from all incident directions. Both the inversion and retention attacking angle distributions decrease steeply at small angles as a sign of the nonreactive (especially for inversion) orientation of the deep halogen-bonded FS premin. The retention S_N_2 attacking-angle distribution is also isotropic at low collision energies; however, at higher energies, these curves show maxima at around 80° attacking angle, reflecting the 82.5° FPI bond angle of the planar FS TS and thereby suggesting a preference for direct front-side-attack.

Dynamics results for the PT channel are shown in Figure 4. The maximum value of the impact parameter, where the reaction probability becomes zero, is the largest (27 bohr) at the lowest energy and decreases significantly, from 16 to 12 bohr, as collision energy increases. The distinct behavior at the lowest collision energy can be spotted for this channel as well. The opacity functions favoring large-*b* collisions and the forward-peaking scattering-angle distributions show an obvious preference for the stripping mechanism at all energies, except the lowest 0.9 kcal/mol collision energy, which exhibits an almost flat isotropic distribution, evidence of complex formation.

**Figure 4:**
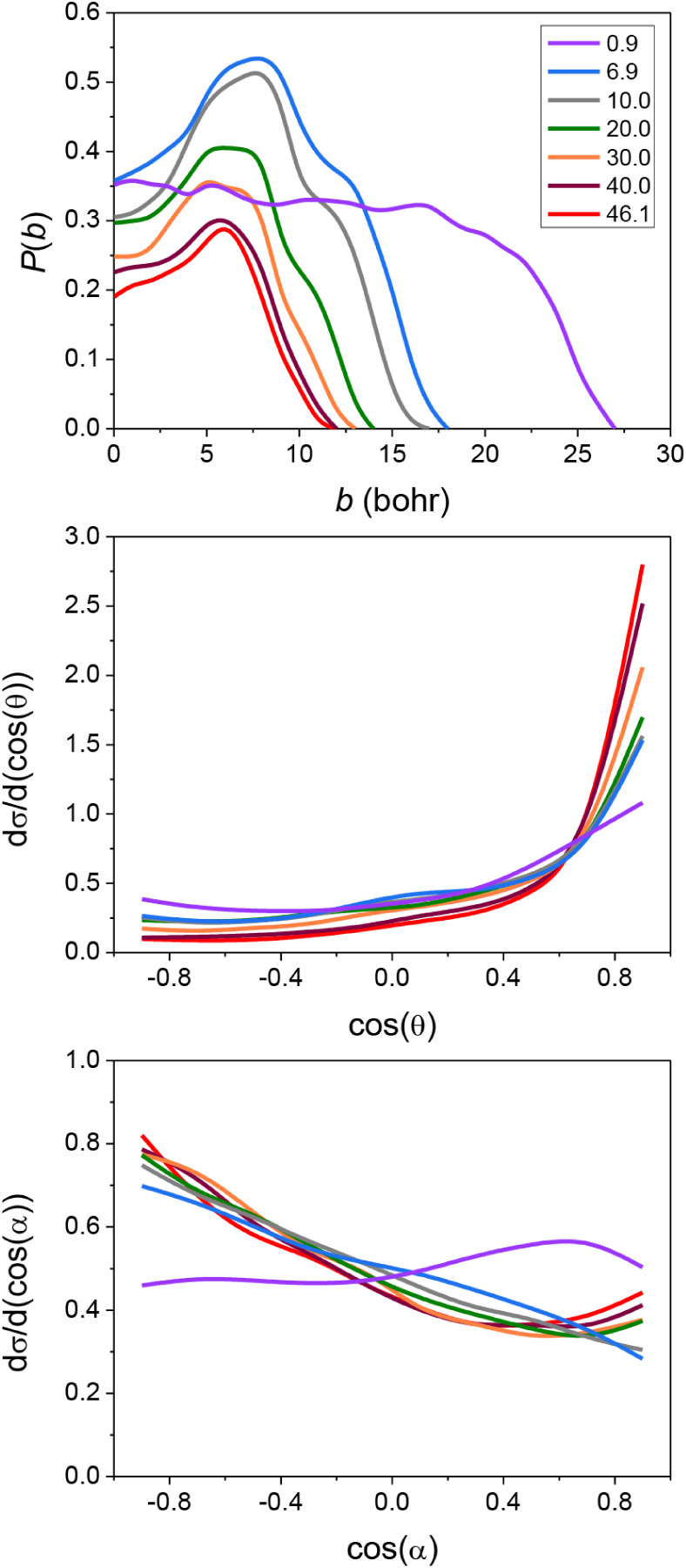
Opacity functions (upper panel), scattering angle distributions (middle panel), and attacking angle distributions (lower panel) of the PT channel of the F^–^ + PH_2_I reaction at different collision energies (kcal/mol) denoted with different colors.

The energy flow during the F^–^ + PH_2_I reaction is also investigated, and the results are shown in Figure 5. The relative translational energy distributions of the products of the S_N_2 reaction are colder than those of the proton-transfer channel. In contrast, the internal degrees of freedom (DoFs) of the S_N_2 product molecule (PH_2_F) are substantially, while the internal motion of the PT products is only moderately excited. In the case of the S_N_2 translational energy distributions, we do not see much collision-energy dependence; however, a part of the translational energy of the reactants can easily convert into product recoil. The initial translational energy is also found to excite the internal DoFs of the PH_2_F product, as the internal-energy maxima blue-shift with increasing collision energy. In comparison, the internal energy distributions of the PHI^–^ and HF products do not show significant dependence on the initial translational energy, while collision energy flows very efficiently into product translation during proton transfer. From the above observations, the S_N_2 reaction seems to be more indirect than the proton-transfer pathway, which is also reflected by the shape of the PH_2_F internal-energy curves that peak around the maximal available energy, indicating the formation of complexes with high internal energy. We also decompose the internal energy of the products into vibrational and rotational energy. From Figure 5, we can see that both the vibrational and the rotational motion of the PH_2_F molecule are excited, with more energy in vibrations, especially at the lowest collision energy. For the PT products, the results show a similar magnitude of vibrational and rotational excitation, with slightly more vibrational energy flowing into HF than into PHI^–^.

**Figure 5:**
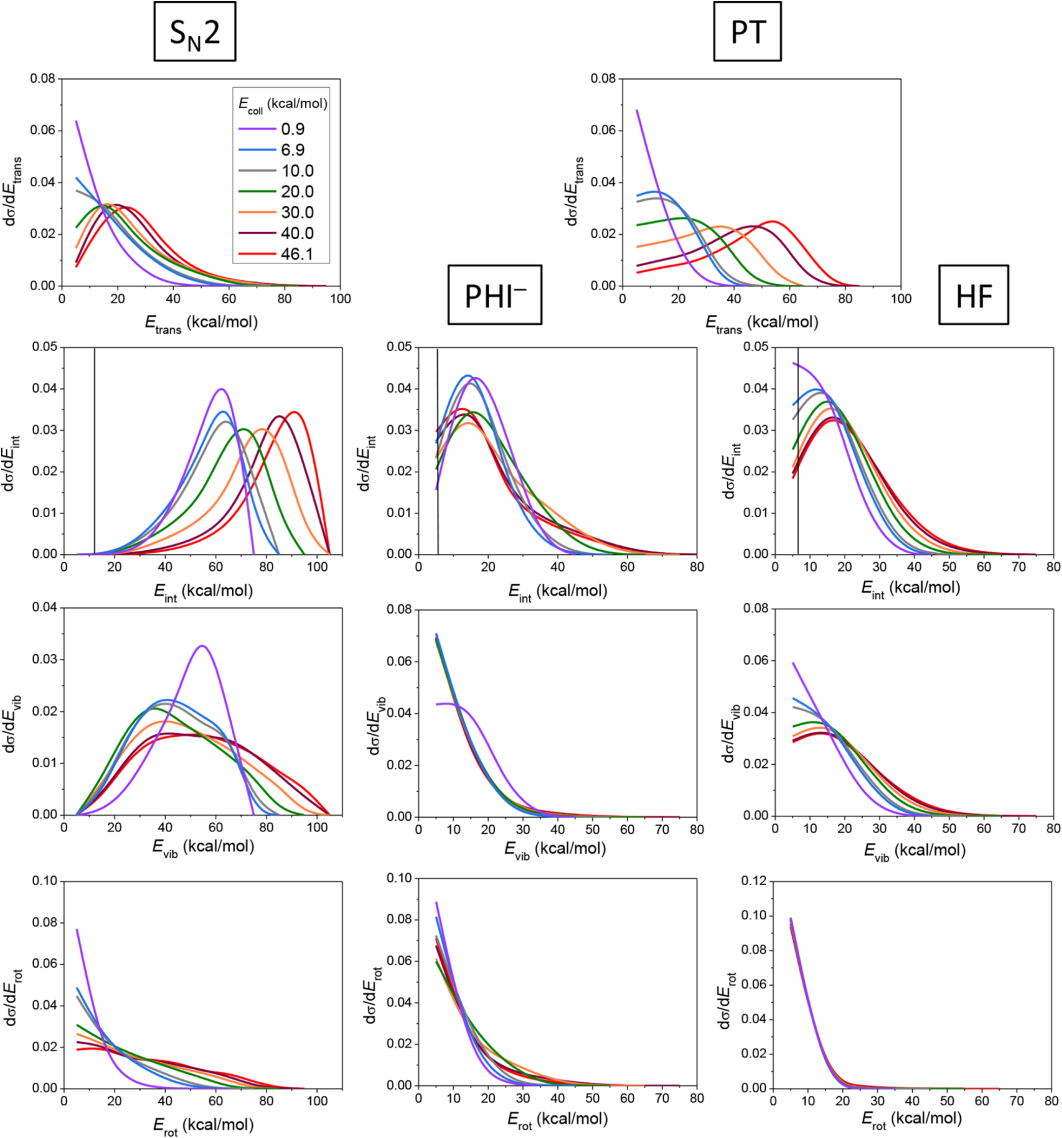
Relative translational energy distributions of the products of the S_N_2 and PT channels (1st row), along with internal energy distributions of the PH_2_F, PHI^–^, and HF products of the F^–^ + PH_2_I reaction (2nd row) decomposed into vibrational (3rd row) and rotational (4th row) energy distributions at different collision energies (kcal/mol) denoted with different colors.

The S_N_2 pathway of the F^–^ + PH_2_I reaction shows similar dynamics to the analogous reaction with the Cl^–^ leaving group in terms of scattering angles; however, in the opacity functions, one may notice an increased ratio of large-*b* stripping with respect to F^–^ + PH_2_Cl.^61^ Regarding the S_N_2 energy distributions, in the case of the title reaction, they suggest somewhat more indirect dynamics at higher collision energies than in its Cl^–^-leaving-group analogue. At phosphorus center, we do not see the strong shift to indirectness observed in the analogous S_N_2@C reactions^17,20^ when changing the Cl^–^ leaving ion to I^–^, because, while the deep FS premin does cause more indirect dynamics in the title reaction, multi-inversion is cut out from the picture here, which in turn is one of the reasons behind the indirect F^–^ + PH_2_Cl dynamics.^61^ The proton-transfer route does not feature marked differences in the case of the two different leaving ions. Nevertheless, a major deviation is seen in the stereodynamics regarding the two leaving groups: the F^–^ + PH_2_I reaction exhibits an increased preference for retention at lower collision energies than F^–^ + PH_2_Cl, which can be explained by the deeper FS premin, which guides the process to choose the front-side attack path leading to the retention of the initial configuration. It should be noted that product configuration-resolved angle and energy distributions have not been investigated in the case of the Cl^–^ leaving group.

A main advantage of QCT simulations is that the trajectories can be easily visualized, which gives deeper insight into the actual mechanisms of the reactions investigated. Due to the main difference being the increased ratio of retention in the F^–^ + PH_2_I reaction with respect to its analogue with the Cl^–^ leaving group, we analyze representative quasi-classical trajectories with S_N_2 products of the title reaction that retained their initial configuration. Regarding the F^–^ + PH_2_Cl reaction, we have identified three possible retention mechanisms at low collision energies: direct front-side, complex-forming multi-inversion, which includes repeated proton-abstraction-induced entrance-channel inversions, and post-Walden double inversion, where the proton-abstraction-induced inversion occurs in the exit channel.^61^ At higher energies, only the direct front-side attack mechanism survives.^61^ In contrast, when the leaving group is changed to I^–^, in low-collision-energy retention trajectories, besides direct front-side attack, we also find pre-Walden double inversion, where the F^–^ changes the configuration around the central atom by abstracting a proton and putting it back later, before the system hits the Walden minimum, similarly to what was observed in the case of carbon center.^16,21^ In contrast to F^–^ + PH_2_Cl, here, this proton-abstraction-induced inversion happens only once, probably due to the larger size of I, thereby “reducing” multiinversion to just double inversion. We also often see the self-inversion of the Walden-inversion product, demonstrating its high vibrational umbrella excitation, which has only rarely been noted for F^–^ + NH_2_Cl,^55^ and turned out to be even less likely for F^–^ + PH_2_Cl.^61^ This might be explained by the higher exothermicity and the smaller energy gap between the products and the Walden minimum in the case of the I^–^ leaving group. A minor route of proton abstraction followed by mounting the FS TS barrier with a planar arrangement is also identified in the title reaction. As the collision energy increases, a new reaction path appears with the first step of hitting the FS premin structure and then proceeding via either the nonplanar FS TS’ or the planar FS TS barrier, supporting the guiding role of FS premin in the observed increasing ratio of retention. Along with this mechanism, we also see direct front-side, post-Walden self- and double-inversion at the highest collision energy for the title reaction, accounting for a more indirect F^–^ + PH_2_I reaction at higher energies relative to F^–^ + PH_2_Cl. The visual inspection of the trajectories also strengthens that at phosphorus center, multi-inversion, a key mechanism at N-center,^55^ is not significant, probably due to the extremely stable Walden-inversion complex, which competes effectively with the much higher-energy H-bonded entrance-channel minimum and TS involved in multi-inversion. Interestingly, “only” double inversion occurs in some cases, presumably owing to the more crowded environment of the P-center due to the large I ligand.

The deep halogen-bonded prereaction minimum causing an increased retention ratio and indirect dynamics in the title reaction is a general feature of the S_N_2 reactions with halide nucleophiles and leaving groups at carbon-,^20^ silicon-,^46^ nitrogen-,^54^ and phosphorus-center,^62^ when the nucleophile is F^–^ and the leaving group is I^–^,^20,46,54,62^ due to the characteristics of the σ-hole interactions, including halogen bonds.^69^ Dynamics studies at carbon center^17,20^ have already showed a significant shift to indirectness with respect to the Cl^–^ leaving group, which, along with the present findings, may imply that such deep halogen-bonded minima may have a similar role in all these reactions.

## Conclusions

Here, we develop a novel high-level ab initio analytical potential energy surface for the F^–^ + PH_2_I reaction and study its dynamics using the quasi-classical trajectory method, with a special focus on the stereodynamics, along with the effect of the I^–^ leaving group by comparing our results to previous ones obtained for the F^–^ + PH_2_Cl reaction.^61^ The energetics of the title reaction are generally more exothermic with respect to F^–^ + PH_2_Cl, with the outstandingly deep halogen-bonded front-side prereaction complex. This stable complex turns out to considerably impact dynamics: on one hand, by an overall inhibiting effect on the S_N_2 reaction and thus the indirect promotion of proton transfer; on the other hand, by guiding a part of reactive S_N_2 collisions toward front-side attack over Walden inversion due to its nonreactive orientation for the latter, thereby increasing the ratio of retention at lower collision energies than what has been observed for the F^–^ + PH_2_Cl reaction. The S_N_2 route is found to be rather indirect, mainly due to the deep halogen-bonded minimum, even more than the analogous reaction with the Cl^–^ leaving group, forming internally highly excited products, with more energy stored in vibration than in rotation. Inversion, the predominant pathway owing to the extremely deep Walden minimum occurs mainly through the stripping mechanism, with backward-scattering direct rebound being less relevant. In contrast, retention is more of a complex-forming pathway that shows a slight sideways preference at higher collision energies, reflecting the increasing prevalence of front-side attack with increasing energy. It turns out that multi-inversion, dominant in the F^–^ + NH_2_Cl reaction,^55^ is not significant in the title reaction; thus, the front-side-attack route is supposed to be the one to primarily provide products with retained configuration. Proton transfer, in contrast to S_N_2, is a direct pathway featuring a clear forward-scattering preference, indicating a prevailing stripping mechanism leading to moderately excited products, with similar amounts of energy in internal rotation and vibration. The qualitative analysis of quasi-classical trajectories reveals various retention mechanisms in this phosphorus-centered S_N_2 reaction, such as direct front-side attack, pre- or post-Walden double inversion, post-Walden self-inversion, and proton-abstraction- or halogen-bond-mediated front-side attack. It seems that double inversion is favored over multi-inversion in the title reaction, possibly because of the large size of the I ligand. We hope that our investigation will contribute to the understanding of the atomic-level governing forces in S_N_2 reactions, highlighting central-atom and leaving-group effects with a particular focus on stereodynamics.

## References

[1] Bohme D. K., Young L. B. (1970). Kinetic Studies of Reactions of Oxide, Hydroxide, Alkoxide,
Phenyl, and Benzylic Anions with Methyl Chloride in Gas Phase at 22.5
Degrees. J. Am. Chem. Soc..

[2] Olmstead W. N., Brauman J. I. (1977). Gas-Phase Nucleophilic
Displacement Reactions. J. Am. Chem. Soc..

[3] Barlow S. E., Van Doren J. M., Bierbaum V. M. (1988). The Gas-Phase Displacement Reaction
of Chloride Ion with Methyl Chloride as a Function of Kinetic Energy. J. Am. Chem. Soc..

[4] Giles K., Grimsrud E. P. (1992). The Kinetic Ion Mobility Mass Spectrometer: Measurements
of Ion–Molecule Reaction Rate Constants at Atmospheric Pressure. J. Phys. Chem..

[5] Glukhovtsev M. N., Pross A., Radom L. (1995). Gas-Phase Identity S_N_2
Reactions of Halide Anions with Methyl Halides: A High-Level Computational
Study. J. Am. Chem. Soc..

[6] Viggiano A. A., Morris R. A. (1996). Rotational and Vibrational
Energy Effects on Ion–Molecule
Reactivity as Studied by the VT-SIFDT Technique. J. Phys. Chem. A.

[7] Ayotte P., Kim J., Kelley J. A., Nielsen S. B., Johnson M. A. (1999). Photoactivation
of the Cl^–^ + CH_3_Br S_N_2 Reaction
via Rotationally Resolved C-H Stretch Excitation of the Cl^–^·CH_3_Br Entrance Channel Complex. J. Am. Chem. Soc..

[8] Sun L., Song K., Hase W. L. (2002). A S_N_2 Reaction That Avoids
Its Deep Potential Energy Minimum. Science.

[9] Gonzales J. M., Pak C., Cox R. S., Allen W. D., Schaefer H. F., Császár A. G., Tarczay G. (2003). Definitive ab Initio
Studies of Model S_N_2 Reactions CH_3_X + F^–^ (X = F, Cl, CN, OH, SH, NH_2_, PH_2_). Chem. – Eur. J..

[10] Mikosch J., Trippel S., Eichhorn C., Otto R., Lourderaj U., Zhang J.-X., Hase W. L., Weidemüller M., Wester R. (2008). Imaging Nucleophilic Substitution Dynamics. Science.

[11] Zhang J., Mikosch J., Trippel S., Otto R., Weidemüller M., Wester R., Hase W. L. (2010). F^–^ + CH_3_I → FCH_3_ + I^–^ Reaction Dynamics.
Nontraditional Atomistic Mechanisms and Formation of a Hydrogen-Bonded
Complex. J. Phys. Chem. Lett..

[12] Manikandan P., Zhang J., Hase W. L. (2012). Chemical
Dynamics Simulations of
X^–^ + CH_3_Y → XCH_3_ +
Y^–^ Gas-Phase S_N_2 Nucleophilic Substitution
Reactions. Nonstatistical Dynamics and Nontraditional Reaction Mechanisms. J. Phys. Chem. A.

[13] Hennig C., Schmatz S. (2012). Differential Reaction Cross Sections from Rotationally
Resolved Quantum Scattering Calculations: Application to Gas-Phase
S_N_2 Reactions. Phys. Chem. Chem.
Phys..

[14] Szabó I., Császár A. G., Czakó G. (2013). Dynamics of
the F^–^ + CH_3_Cl → Cl^–^ + CH_3_F S_N_2 Reaction on a Chemically Accurate
Potential Energy Surface. Chem. Sci..

[15] Xie J., Otto R., Mikosch J., Zhang J., Wester R., Hase W. L. (2014). Identification of
Atomic-Level Mechanisms for Gas-Phase
X^–^ + CH_3_Y S_N_2 Reactions by
Combined Experiments and Simulations. Acc. Chem.
Res..

[16] Szabó I., Czakó G. (2015). Revealing
a Double-Inversion Mechanism for the F^–^ + CH_3_Cl S_N_2 Reaction. Nat. Commun..

[17] Stei M., Carrascosa E., Kainz M. A., Kelkar A. H., Meyer J., Szabó I., Czakó G., Wester R. (2016). Influence of the Leaving
Group on the Dynamics of a Gas-Phase S_N_2 Reaction. Nat. Chem..

[18] Xie J., Hase W. L. (2016). Rethinking the S_N_2 Reaction. Science.

[19] Li Y., Wang Y., Wang D. Y. (2017). Quantum Dynamics Study of the Potential
Energy Minima Effect on Energy Efficiency for the F^–^ + CH_3_Cl → FCH_3_ + Cl^–^ Reaction. J. Phys. Chem. A.

[20] Szabó I., Olasz B., Czakó G. (2017). Deciphering
Front-Side Complex Formation
in S_N_2 Reactions via Dynamics Mapping. J. Phys. Chem. Lett..

[21] Szabó I., Czakó G. (2017). Dynamics and Novel Mechanisms of
S_N_2 Reactions
on ab Initio Analytical Potential Energy Surfaces. J. Phys. Chem. A.

[22] Tasi D. A., Fábián Z., Czakó G. (2019). Rethinking
the X^–^ + CH_3_Y [X = OH, SH, CN, NH_2_, PH_2_; Y = F, Cl, Br, I] S_N_2 Reactions. Phys. Chem. Chem. Phys..

[23] Tasi D. A., Győri T., Czakó G. (2020). On the Development of a Gold-Standard
Potential Energy Surface for the OH^–^ + CH_3_I Reaction. Phys. Chem. Chem. Phys..

[24] Bastian B., Michaelsen T., Li L., Ončák M., Meyer J., Zhang D. H., Wester R. (2020). Imaging Reaction Dynamics
of F^–^(H_2_O) and Cl^–^(H_2_O) with CH_3_I. J. Phys. Chem.
A.

[25] Meyer J., Tajti V., Carrascosa E., Győri T., Stei M., Michaelsen T., Bastian B., Czakó G., Wester R. (2021). Atomistic Dynamics of Elimination and Nucleophilic
Substitution Disentangled for the F^–^ + CH_3_CH_2_Cl Reaction. Nat. Chem..

[26] Tasi D. A., Czakó G. (2021). Uncovering
an Oxide Ion Substitution for the OH^–^ + CH_3_F Reaction. Chem. Sci..

[27] Wester R. (2022). Fifty Years
of Nucleophilic Substitution in the Gas Phase. Mass Spectrom. Rev..

[28] Qin J., Liu Y., Li J. (2022). Quantitative Dynamics of Paradigmatic S_N_2 Reaction OH^–^ + CH_3_F on Accurate Full-Dimensional
Potential Energy Surface. J. Chem. Phys..

[29] Lu X., Shang C., Li L., Chen R., Fu B., Xu X., Zhang D. H. (2022). Unexpected Steric Hindrance Failure in the Gas Phase
F^–^ + (CH_3_)_3_CI S_N_2 Reaction. Nat. Commun..

[30] Zhao S., Fu G., Zhen W., Wang H., Liu M., Yang L., Zhang J. (2023). Nucleophile Effects on the E2/S_N_2 Competition for the
X^–^ + CH_3_CH_2_Cl Reactions: A
Theoretical Study. J. Phys. Chem. A.

[31] Fu G., Zhen W., Wang H., Yang L., Zhang J. (2024). The Investigation
on the Reactivity and the Formation of Halogen Bond Complexes for
the Reactions of α-Nucleophiles XO^–^ (X = F,
*Cl, Br, I) and CH_3_CH_2_Cl. J. Phys. Chem. A.

[32] Zhu Y., Li Y., Wang D. (2024). Potentials of Mean Force and Solvent
Effects of the
CN^–^ + CH_3_X (X = F, Cl, Br, and I) Reactions
by the N-Side Attack in Aqueous Solution. J.
Phys. Chem. A.

[33] Ayasli A., Khan A., Gstir T., Michaelsen T., Papp D., Wang Y., Song H., Yang M., Czakó G., Wester R. (2025). A Dynamic Isotope Effect in the Nucleophilic
Substitution Reaction between F^–^ and CD_3_I. Nat. Commun..

[34] Liu X., Jia M., Tian S., Li H., Pang B., Wu Y. (2025). Competition
between S_N_2 and E2 Pathways in CN^–^ +
RI/RF Systems: Effects of Reactive Centers, Substitution, and Leaving
Groups. J. Phys. Chem. A.

[35] Wu X., Zhu C., Francisco J. S., Xie J. (2025). Single Solvent Molecule Effect over
S_N_2 and E2 Competition in the Hydroperoxide Anion Reaction
with Ethyl-Iodide. Chem. Sci..

[36] Gutal A., Paranjothy M. (2025). Quasi-Classical
Trajectory Study of the CN^–^ + CH_3_I Reaction
on a High-Dimensional Neural Network
PES. J. Chem. Phys..

[37] Zhen W., Fu G., Yang L., Wang H., Sheng L., Sun J., Zhang J. (2025). Dynamics of Sterically Hindered F^–^ + *i*-C_3_H_7_Cl Reaction: An Enhancement of Indirect
Mechanisms. J. Chem. Phys..

[38] Walden P. (1896). Ueber die
Gegenseitige Umwandlung Optischer Antipoden. Ber. Dtsch. Chem. Ges..

[39] Ingold, C. K. Structure and Mechanisms in Organic Chemistry; Cornell University Press: Ithaca, NY, 1953.

[40] Bento A. P., Bickelhaupt F. M. (2007). Nucleophilic
Substitution at Silicon (S_N_2@Si) via a Central Reaction
Barrier. J. Org.
Chem..

[41] van
Bochove M. A., Bickelhaupt F. M. (2008). Nucleophilic Substitution at C, Si
and P: How Solvation Affects the Shape of Reaction Profiles. Eur. J. Org. Chem..

[42] Yang Z.-Z., Ding Y.-L., Zhao D.-X. (2009). Theoretical Analysis of Gas-Phase
Front-Side Attack Identity S_N_2­(C) and S_N_2­(Si)
Reactions with Retention of Configuration. J.
Phys. Chem. A.

[43] Hupf E., Olaru M., Rat C. I., Fugel M., Hübschle C. B., Lork E., Grabowsky S., Mebs S., Beckmann J. (2017). Mapping the
Trajectory of Nucleophilic Substitution at Silicon Using a *peri*-Substituted Acenaphthyl Scaffold. Chem. – Eur. J..

[44] Hamlin T. A., Swart M., Bickelhaupt F. M. (2018). Nucleophilic Substitution (S_N_2): Dependence on Nucleophile, Leaving Group, Central Atom,
Substituents, and Solvent. ChemPhysChem.

[45] Fugel M., Dittmer A., Kleemiss F., Grabowsky S. (2021). On the Role
of Hydrogen Bonding in Gas-Phase S_N_2 Reactions at Silicon. J. Phys. Chem. A.

[46] Dékány A. Á., Kovács G. Z., Czakó G. (2021). High-Level
Systematic ab Initio Comparison of Carbon- and Silicon-Centered S_N_2 Reactions. J. Phys. Chem. A.

[47] Dékány A. Á., Czakó G. (2023). Exploring the Versatile Reactivity of the F^–^ + SiH_3_Cl System on a Full-Dimensional Coupled-Cluster
Potential Energy Surface. J. Chem. Phys..

[48] Molnár B. J., Dékány A. Á., Czakó G. (2024). Automated
Potential Energy Surface Development and Quasi-Classical Dynamics
for the F^–^ + SiH_3_I System. J. Chem. Phys..

[49] Bühl M., Schaefer H. F. (1993). S_N_2 Reaction at Neutral
Nitrogen: Transition State Geometries and Intrinsic Barriers. J. Am. Chem. Soc..

[50] Gareyev R., Kato S., Bierbaum V. M. (2001). Gas Phase Reactions of NH_2_Cl with Anionic Nucleophiles: Nucleophilic Substitution at Neutral
Nitrogen. J. Am. Soc. Mass Spectrom..

[51] Lv J., Zhang J., Wang D. (2016). A Multi-Level
Quantum Mechanics and
Molecular Mechanics Study of S_N_2 Reaction at Nitrogen:
NH_2_Cl + OH^–^ in Aqueous Solution. Phys. Chem. Chem. Phys..

[52] Liu X., Zhao C., Yang L., Zhang J., Sun R. (2017). Indirect Dynamics
in S_N_2@N: Insight into the Influence of Central Atoms. Phys. Chem. Chem. Phys..

[53] Kubelka J., Bickelhaupt M. F. (2017). Activation Strain Analysis of S_N_2 Reactions
at C, N, O, and F Centers. J. Phys. Chem. A.

[54] Hajdu B., Czakó G. (2018). Benchmark
ab Initio Characterization of the Complex
Potential Energy Surfaces of the X^–^ + NH_2_Y [X,Y= F, Cl, Br, I] Reactions. J. Phys. Chem.
A.

[55] Papp D., Czakó G. (2021). Facilitated Inversion Complicates the Stereodynamics
of an S_N_2 Reaction at Nitrogen Center. Chem. Sci..

[56] Dutta S. S., Lourderaj U. (2024). Computational
Studies of Nucleophilic Substitution
at Nitrogen Center: Reactions of NH_2_Cl with HO^–^, CH_3_O^–^ and C_2_H_5_O^–^. ChemPhysChem.

[57] Dutta S. S., Lourderaj U. (2025). Unconventional Pathways in Nitrogen-Centered
S_N_2 Reactions: From Roundabout to Hydride Transfer. J. Phys. Chem. A.

[58] van
Bochove M. A., Swart M., Bickelhaupt F. M. (2006). Nucleophilic
Substitution at Phosphorus (S_N_2@P): Disappearance and Reappearance
of Reaction Barriers. J. Am. Chem. Soc..

[59] van
Bochove M. A., Swart M., Bickelhaupt F. M. (2007). Nucleophilic
Substitution at Phosphorus Centers (S_N_2@P). ChemPhysChem.

[60] Kolodiazhnyi O. I., Kolodiazhna A. (2017). Nucleophilic Substitution at Phosphorus: Stereochemistry
and Mechanisms. Tetrahedron: Asymmetry.

[61] Giricz A., Czakó G., Papp D. (2023). Alternating Stereospecificity upon
Central-Atom Change: Dynamics of the F^–^ + PH_2_Cl S_N_2 Reaction Compared to its C- and N-Centered
Analogues. Chem. – Eur. J..

[62] Ballay B., Szűcs T., Papp D., Czakó G. (2023). Phosphorus-Centered
Ion–Molecule Reactions: Benchmark ab Initio Characterization
of the Potential Energy Surfaces of the X^–^ + PH_2_Y [X, Y= F, Cl, Br, I] Systems. Phys.
Chem. Chem. Phys..

[63] Győri T., Czakó G. (2020). Automating the Development of High-Dimensional Reactive
Potential Energy Surfaces with the Robosurfer Program System. J. Chem. Theory Comput..

[64] Győri T., Czakó G. (2022). ManyHF: A Pragmatic Automated Method
of Finding Lower-Energy
Hartree–Fock Solutions for Potential Energy Surface Development. J. Chem. Phys..

[65] Werner, H.-J. ; Knowles, P. J. ; Knizia, G. ; Manby, F. R. ; Schütz, M. ; Celani, P. ; Györffy, W. ; Kats, D. ; Korona, T. ; Lindh, R. ; Molpro, version 2015.1, A package of ab initio programs; http://www.molpro.net.

[66] Braams B. J., Bowman J. M. (2009). Permutationally Invariant Potential
Energy Surfaces
in High Dimensionality. Int. Rev. Phys. Chem..

[67] Szűcs T., Czakó G. (2024). Automated
Potential Energy Surface Development and
Comprehensive Dynamics for the F + CH_3_NH_2_ Reaction. J. Chem. Phys..

[68] Hase, W. L. Encyclopedia Of Computational Chemistry; Wiley: New York, 1998, pp. 399–407.

[69] Politzer P., Murray J. S., Clark T. (2013). Halogen Bonding and Other σ-Hole
Interactions: A Perspective. Phys. Chem. Chem.
Phys..

